# Recent Advances in Optical Biosensors for Environmental Monitoring and Early Warning

**DOI:** 10.3390/s131013928

**Published:** 2013-10-15

**Authors:** Feng Long, Anna Zhu, Hanchang Shi

**Affiliations:** 1 School of Environment and Natural Resources, Renmin University of China, No.59, Zhongguancun Street, Haidian District, Beijing 100872, China; 2 Research Institute of Chemical Defence, No.1, Huanyin Street, Changping District, Beijing 100872, China; E-Mail: zhuanna00@mails.thu.edu.cn; 3 State Key Joint Laboratory of ESPC, School of Environment, Tsinghua University, No.1, Tsinghua Yuan, Haidian District, Beijing 100872, China

**Keywords:** optical biosensor, environmental pollution control, nanosensor, biomolecules

## Abstract

The growing number of pollutants requires the development of innovative analytical devices that are precise, sensitive, specific, rapid, and easy-to-use to meet the increasing demand for legislative actions on environmental pollution control and early warning. Optical biosensors, as a powerful alternative to conventional analytical techniques, enable the highly sensitive, real-time, and high-frequency monitoring of pollutants without extensive sample preparation. This article reviews important advances in functional biorecognition materials (e.g., enzymes, aptamers, DNAzymes, antibodies and whole cells) that facilitate the increasing application of optical biosensors. This work further examines the significant improvements in optical biosensor instrumentation and their environmental applications. Innovative developments of optical biosensors for environmental pollution control and early warning are also discussed.

## 1. Introduction

Innovative analytical devices featuring precision, sensitivity, specificity, speed, and usability continue to be developed to meet the increasing demand for legislative actions on the monitoring of a growing number of pollutants. To detect different environmental contaminants, quantitative analysis of water samples is generally performed with traditional analytical methods such as chromatographic and spectroscopic technologies. Although accurate and sensitive, these methods require sophisticated and expensive instrumentation, expert personnel for their operation, and multistep and complicated sample preparation. These methods are also labour intensive and time consuming, and it is difficult to achieve on-site, real-time, and high-frequency monitoring of contaminants [1]. To meet these requirements, researchers have been striving to develop robust, cost-effective, automated water-monitoring devices for the rapid and sensitive analysis of environmental pollutants. Combining biochemistry, biology, nanotechnology, physics, and electronics, biosensors can follow new developments in the introduction of risk assessment/management approaches and environmental legislation because of their unique characteristics such as speed, sensitivity, specificity, ease-of-use, and real-time remote monitoring capability [2].

**Figure 1.**
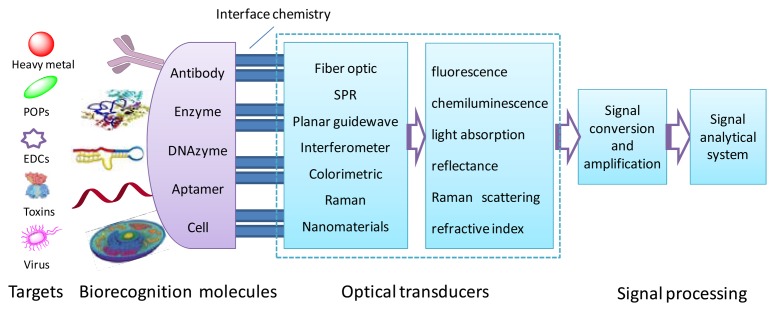
Schematic of an optical biosensor.

A biosensor is an analytical device that integrates a biological sensing element (e.g., an enzyme or aqn antibody) with a physical (e.g., optical, mass, or electrochemical) transducer, whereby the interaction between the target and the bio-recognition molecules is translated into a measurable electrical signal [3]. Optical biosensors that exploit light absorption, fluorescence, luminescence, reflectance, Raman scattering and refractive index are powerful alternatives to conventional analytical techniques (Figure 1). These biosensors provide rapid, highly sensitive, real-time, and high-frequency monitoring without any time-consuming sample concentration and/or prior sample pre-treatment steps. Although optical biosensors have great potential applications in the areas of environmental monitoring, food safety, drug development, biomedical research, and diagnosis [4–10], their use in fields of environmental pollution control and early warning is still in the early stages.

Tremendous progress has been achieved in the development of optical biosensors, and numerous research papers and outstanding reviews were published in the literature in recent years [2,5–11]. This review focuses on recent advancements in optical biosensors and provides examples of relevant, specific applications and their analytical performance in environmental pollution monitoring and early warning. Bio-recognition molecules are essential to biosensing and are highlighted first. The significant improvements in optical biosensor instrumentation will then be discussed. Finally, new developments in optical biosensors for pollution control and early warning will be reviewed.

## 2. Biorecognition Molecules

The fundamental and key feature of a biosensor is the construction of the bio-recognition element for the interaction with the targets. Functional biomaterials with high affinity and high specificity include antibodies, enzymes, functional oligonucleotides and whole cells [5,9–12].

### 2.1. Enzymes

Enzymes are substrate-specific biological molecules that catalyze specific chemical reactions. Enzyme-based optical biosensors have been extensively studied in the last decades due to the vital practical needs of industry, medicine, and environmental control and monitoring [4,11,13]. The immobilization of enzymes on solid substrates is extremely important because the immobilisation method can enhance the working lifetime and sensitivity of the biosensors. The optical transducers of enzyme-based biosensors are at the heart of the development of compact, self-contained devices for environmental monitoring. Cholinesterase (ChE) enzymes can be inhibited by several toxic chemicals such as organophosphates and pesticides, heavy metals, and toxins. Thus, ChE biosensors are of particular interest in the area of global toxicity monitoring [4,14,15]. Considering that different pollutants inhibit enzyme activity in various ways, multi-analyte detection can be achieved using enzyme sensors. For example, pesticides and heavy metal ions can be detected simultaneously in a sample solution through the inhibition of butyrylcholine esterase by pesticides and urease by heavy metals ions [4–6,16].

An enzymatic biosensor for the measurement of toluene in aqueous solutions was constructed and characterized [17]. Toluene *ortho*-monooxygenase was used as biorecognition element, and an oxygen-sensitive ruthenium-based phosphorescent dye served as transducer. Toluene was determined based on the enzyme-catalyzed consumption of oxygen that changed the phosphorescence intensity of the oxygen-sensitive probe. Although the enzymatic biosensor can detect toluene in wastewater with a limit of detection (LOD) of 3 μM and a linear signal range up to 100 μM, the response time is long (1 h), and the activity decreases with each measurement and with storage time. Huang *et al.* developed a fiber optic biosensor for the determination of adrenaline based on immobilized laccase catalysis [18]. The laccase-containing nanoparticle and the luminescent oxygen-sensing membrane were deposited at the tip of an optical fiber. The enzyme laccase catalyzes the oxidation of adrenaline through oxygen consumption. The biosensor can detect adrenaline ranging from 10 nM to 1 μM concentrations with a typical response time of 30 s. The immobilized enzyme is fairly stable.

Enzyme-based optical biosensors open novel ways of performing the rapid, remote, in-line determinations for environmental pollution control and early warning. Despite the fact that great progress has been made in improving the reliability of enzyme-based optical biosensors and extending their capabilities to higher sensitivity and selectivity and faster response time, a number of limitations still exist in environmental pollution control and early warning [11]. First, a limited number of substrates have been evolved for their specific enzymes; Second, the interaction between environmental pollutants and specific enzymes is relatively limited; Third, the enzymes lack specificity in terms of differentiating among compounds of similar classes [6,16].

### 2.2. Antibodies

Using the specific interactions between antigen and antibody, immunosensors have been regarded as the gold-standard technique in environmental monitoring and clinical diagnostics [2,4–7,11]. The highly specific interaction of the two binding sites of an antibody with one particular target can be detected by a transducer (e.g., optical or electronic) [2,5–7]. Therefore, the immunosensor provides a highly repeatable and highly specific reaction format, enabling it to recognize specific environmental contaminants.

Non-immunogenic environmental pollutants with low molecular weights (<1 kDa), called haptens, eventually become immunogenic upon conjugation to carrier proteins [19,20]. Antibodies against haptens, such as pesticides, persistent organic pollutants (POPs), and endocrine disrupting chemicals (EDCs), are prepared by synthesizing immunogens from the covalent binding of the hapten to a carrier protein and then immunizing them into animals. The specificity and quality of antibody, which is important for immunoassay, is mostly determined by the product of the chemical binding of the hapten to the carrier protein, called complete antigen [5]. In order to detect the microcystin-LR (MC-LR), that is the most frequent and most toxic hepatotoxin, the corresponding complete antigen (MC-LR-BSA) was synthesized by introducing a primary amino group in the seventh N-methyldehydroalanine residue of MC-LR [19]. The product aminoethyl-MC-LR was then coupled to bovine serum albumin (BSA) with glutaraldehyde. A monoclonal antibody (Clone MC8C10) against MC-LR was produced by immunization with MC-LR-BSA. An indirect competitive enzyme-linked immunosorbent assay (ic-ELISA) with MC8C10 was established to detect MCs in waters, showing high specificity with a detection limit of 0.1 μg/L for MC-LR [19].

Immunosensors are better than other immunological methods (e.g., ELISA formats) in terms of regeneration and binding properties of the sensing surface, which is critical for the successful reuse of the same sensor surface and the accuracy of detection results [21]. Environmental pollutants are usually small molecular weight substances (molecular weight <1 kDa), and are difficult to directly immobilize onto the biorecognition sensing surface, therefore, antibodies are generally immobilized in the preparation of the sensing surface of immunosensors [5–7,11,21]. However, control over the number, orientation, and position of antibodies relative to the sensor surface is very difficult. Inadvertent disruption of the binding site may occur when the antibody conjugates with the active sensor surface, thus resulting in the inevitable loss of antibody activity [22,23]. Most importantly, the use of strong acid in the regeneration process reduces the recognition capability of immobilized antibodies after sensor surface reuse, thereby affecting the stability and reliability of the immunosensor. Regeneration can be performed no more than 15 times, and in each cycle, antibody activity decreases, which may yield inaccurate detection results [21]. Therefore, hapten-carrier-protein conjugates as bio-recognition molecules were immobilized onto the surface of an immunosensor to obtain a stable reusable sensor. For example, a reusable immunosurface is formed via the covalent attachment of MC-LR-OVA to a self-assembled monolayer generated onto the fiber optic sensor with a heterobifunctional reagent [24]. The regeneration of the sensor surface enables the performance of more than 100 assay cycles devoid of any significant loss of reactivity (less than 5% decrease).

### 2.3. Aptamers

An aptamer, a single-stranded DNA or RNA sequence selected by Systematic Evolution of Ligands by EXponential enrichment (SELEX), binds selectively to its target through folding into a complex three-dimensional structure [4–6,25,26]. The interaction between the aptamer and the target includes structure compatibility, stacking of aromatic rings, electrostatic and van der Waals interactions, hydrogen bonding, or a combination of all these effects [4–6]. Aptamers are a useful alternative to antibodies as sensing molecules, thus introducing a new era of affinity biosensing because of their unique character. Aptamers to target small organic and inorganic compounds such as proteins, peptides, amino acids, nucleotides, drugs, and heavy metal ions can be produced [25–44]. Aptamers can easily be chemically synthesized, and require no complicated and expensive purification steps, which eliminates the batch-to-batch variation found when using antibodies. Furthermore, aptamers can be further modified through chemical synthesis to enhance the stability, affinity, and specificity of the molecules. In addition, aptamers are more stable, and more resistant to denaturation and degradation than antibodies [28,30].

DNA/RNA aptamers intended for POPs, EDCs, organophosphorus pesticides, antibiotics, biotoxins, and pathogenic microorganisms [27–44] are listed in Table 1. Aptamers have become increasingly important bioassay materials for environmental detection.

A reusable evanescent wave aptamer-based biosensor was reported for rapid, sensitive and highly selective detection of 17-*β*-estradiol, a natural endocrine disrupting compound (EDC) with a high estrogenic activity [45]. *β*-Estradiol 6-(O-carboxymethyl)oxime-BSA was covalently immobilized onto the optical fiber sensor surface. The dose-response curve of 17-*β*-estradiol was established with a detection limit of 2.1 nM. The high specificity and selectivity of the sensor were demonstrated by evaluating its response to a number of potentially interfering endocrine-disrupting compounds or other chemicals. Potential interference of real environmental sample matrices was assessed using spiked samples in several tertiary wastewater effluents. This system can be potentially applied for on-site real-time monitoring of 17-*β*-estradiol in wastewater treatment effluents or water bodies.

Several DNA aptamer fluorescence-based sensors have been developed for the detection of Hg^2+^, Pb^2+^, and other trace pollutants [46–49]. Kim *et al.* developed a high-affinity DNA aptamer for arsenic that can bind to arsenate [(As(V)] and arsenite [As(III)] with a dissociation constant of 5 and 7 nM, respectively [41]. Using this aptamer, a colorimetric and resonance scattering (RS)-based biosensor for the ultrasensitive detection of As(III) in aqueous solution via aggregation of gold nanoparticles (AuNPs) by the special interactions between arsenic-binding aptamer, target and cationic surfactant was established [48]. The variations of absorbance and RS intensity were exponentially related to the concentration of As(III) in the range from 1 to 1500 ppb, with the detection limit of 0.6 ppb for colorimetric assay and 0.77 ppb for RS assay.

An aptamer-based fluorescent biosensor was reported for the highly selective and sensitive detection of Pb^2+^ and Hg^2+^ using a G-rich ssDNAs [47], which was labeled with the donor FAM at one end and the quencher DABCYL at the other end. This aptamer has a random-coil structure that changes into a G-quartet structure and a hairpin-like structure upon binding of Pb^2+^ and Hg^2+^, respectively, moving the fluorophore closer to the quencher and resulting in the decrease of the fluorescence intensity. The limits of detection of Pb^2+^ and Hg^2+^ ions are 0.3 nM and 5.0 nM, respectively. Although a variety of aptamers have been successfully selected for environmental contaminants, the detection of real water samples using the appropriate aptamer is still a work in progress.

### 2.4. DNAzymes

DNAzymes (catalytic DNAs or deoxyribozymes) are functional nucleic acids which can fold into a well-defined three-dimensional structure to bind to specific targets [49–52]. DNAzymes can generally obtained through *in vitro* selection, allowing them to function in the presence of a specific target of choice. Combining these DNAzymes that can perform chemical modifications on nucleic acids, with aptamers that can bind with a broad range of molecules generates a new class of functional nucleic acids known as allosteric DNAzymes or aptazymes [49]. The combined specificity of nano-biological recognition probes and the sensitivity of laser-based optical detection allow these DNAzymes to provide unambiguous identification and accurate quantification of environmental pollutants, ranging from low-molecular-weight organic or inorganic substrates and macromolecules to metal ions [50–52]. RNA-cleaving DNAzymes are extensively applied because of their simple reaction conditions, fast turnover rates, and significant possible modifications of their substrate lengths [50].

The high selectivity of DNAzymes toward specific targets makes them ideal biorecognition molecules for biosensing. Numerous DNAzyme-based optical biosensors have been developed for the detection of various heavy metal ions, such as Mg^2+^, Ca^2+^, Zn^2+^, Pb^2+^, Cu^2+^, Co^2+^, Mn^2+^, UO_2_^2+^, Hg^2+^, and Ag^+^ because of their facile operation, high sensitivity, and easily detectable signals [49–52]. Given the tremendous advances made in the areas of functional DNA and nanotechnology, DNAzymes and aptazymes have already been applied to almost every aspect of DNA nanotechnology, resulting in new materials and devices that may be employed in the environmental monitoring field [11,49–52].

### 2.5. Whole Cells

Whole cells are excellent indicators of toxic compounds. A large number of microbial-based optical biosensors have been developed to detect toxicity and pollutants by measuring bioluminescent light production or fluorescence [53]. Olaniran *et al.* developed whole-cell bacterial biosensors for the rapid and effective monitoring of heavy metals and inorganic pollutants in wastewater [53]. Using *Shigella sonnei* and *Escherichia coli*, the biosensors were found to be sensitive to the toxicity of wastewater effluents. Bioluminescence increased with increasing concentration of heavy metals and inorganic pollutants in water with a correlation coefficient (r_2_) as high as 0.995 and 0.997, respectively. These bacterial biosensors are capable of achieving the rapid, sensitive and cost effective detection of wastewater quality.

Arain *et al.* reported an integrated fluorescence-based sensor for pH and oxygen [54], in which bacterial respiratory activity was monitored via the decrease in the oxygen partial pressure of the closed system and also via the decrease in pH value. The inhibitory effect of toxic metal ions on the cellular activity of *E. coli* and *Pseudomonas putida* was then detected. Amaro *et al.* reported a whole-cell biosensor for the detection of heavy metals based on metallothionein promoters from *Tetrahymena thermophila* [55]. Two gene constructs using the *Tetrahymena thermophila* MTT1 and MTT5 metallothionein promoters linked with the eukaryotic luciferase gene, regarded as a reporter. This kind of biosensor appears to be the most sensitive eukaryotic metal biosensor among other published cell biosensors. Using bioluminescent bacteria immobilized in an alginate matrix on the bottom of the wells in a 96-well microplate, Eltzov *et al.* developed a fiber-optic biosensor for monitoring air toxicity [56]. Bioluminescence was suppressed when the biosensor was exposed to toxic compounds present in air Chloroform could be detected by this method with a LOD of 6.6 ppb. The same group developed a flow-through fiber-optic sensing system by immobilizing two other bacterial strains for the online monitoring of toxic pollutants in water [57]. The sensor could detect pollutants in flowing tap water and surface water within 24 h, but a loss of functionality of the bacteria was observed after longer periods.

## 3. Nanomaterials

Nanomaterials exhibit unique size-tunable and shape-dependent physicochemical properties and have numerous possible applications in biosensors [58,59]. The integration of nanomaterials and functional biological molecules (e.g., antibodies, nucleic acids, peptides) opens a new era in the optical biosensor field.

### 3.1. Quantum Dots

Quantum dots (QDs), light-emitting semiconductor nanorystals, have been increasing used as biomolecular detection tools because of their unique optical properties, which conferred advantages over traditional fluorophores such as organic dyes [59,60]. QDs have found applications ranging from bioanalytical assays, to live cell imaging, fixed cell and tissue labeling, and biosensors. The narrow, size-tuned, and symmetric emission spectra of QDs have made them excellent donors for fluorescence resonance energy transfer (FRET) sensors. Moreover, the overlap between the emission spectra of the donor and acceptor is reduced, and the cross-talk in such FRET pairs is circumvented [59–61]. The broad excitation spectra of QDs facilitate excitation at a single wavelength far removed (>100 nm) from their respective emissions, allowing QDs to be used in multiplex assays with single excitation sources. Using covalent or non-covalent linking approaches, the surface modification of QDs with antibodies, aptamers, and peptides are the most developed and widespread detection bioprobes. The long-term photostability, superior brightness, and good chemical stability of QDs enable them to greatly improve bioassay sensitivities and limits [61–63].

However, controlling the number of antibodies (or aptamers) per QD as well as their orientation and position relative to the QD is difficult. Given the possibility of the inadvertent disruption of the binding site when QD conjugates with the antibody, the activity loss of the antibody is inevitable [60,64]. Additionally, antibodies usually need to be cryopreserved, but QDs cannot be frozen, thus making the storage of the QD-antibody a major obstacle to its practical application.

A carrier-protein-hapten-coupled QD nanobioprobe protocol has been developed to perform rapid and sensitive detection of small targets in environmental samples [65]. The determination of 2,4-D in aqueous media was performed by grafting haptens-BSA conjugate on QDs and using the resulting material as a nano-bioprobe for 2,4-D biosensing. Samples containing different concentrations of 2,4-D were mixed with a given concentration of QD immunoprobe and fluorescence-labeled antibody, after which they were competitively detected by the all-fiber microfluidic biosensing platform. A higher concentration of 2,4-D resulted in less fluorescence-labeled anti-2,4-D antibody bound to the QD immunoprobe surface and consequently, lower fluorescence signal [65]. The quantification of 2,4-D over concentration ranges from 0.5 nM to 3 μM with a LOD of 0.5 nM. The method combined the merits of specific and stable binding interactions between environmental pollutants and its specific antibody, as well as the excellent photophysical properties of QDs. The proposed immunosensor had the following unique advantages: first, QD-BSA-haptens conjugates used as recognition elements prevent compromise among the binding properties of the immobilized biomolecules (e.g., antibodies and enzymes); second, the binding sites of QD-BSA-haptens avoided steric hindrance and retained their high activity for their antibody; third, the structure of the QD-BSA-haptens conjugate was more stable in complex environmental samples than typical biorecognition molecules (e.g., antibody and enzymes). The FRET efficiency was higher because of the more abundant acceptor dyes bound to one QD surface, which conferred the QD-FRET assay with high sensitivity. This will provide a universal approach using a QD-bioconjugate as a nano-bioprobe to construct practical FRET-based immunoassay of various small molecules and other applications.

### 3.2. Gold Nanoparticles

Gold nanoparticles (GNPs) with controlled geometrical, optical, and surface chemical properties have great potential applications in environmental and medical detection. GNPs can be easily modified with biomolecules. GNP-based optical biosensors commonly utilize fluorescence quenching through FRET or a visible color change attributed to the aggregation of AuNPs of appropriate sizes [66]. GNP-based optical sensors have been used to detect environmental pollutants including heavy metals, toxins, and other pollutants [66].

Heavy metal contaminations have greatly attracted public attention worldwide because of their serious negative health effects. Liu *et al.* [67] used quaternary ammonium-functionalized GNPs to devise a colorimetric sensor for Hg^2+^ detection with the abstraction of GNP stabilizing thiols by Hg^2+^ inducing aggregation. An AuNP-rhodamine 6G-based fluorescent sensor was used to detect Hg^2+^, which had a LOD of 0.012 ppb [68]. A T-Hg^2+^-T structure was used to develop a detection method of aqueous Hg^2+^ with a LOD of 50 nM [69], in which specific interaction of Hg^2+^ with thymine residues from two AuNPs induces the aggregation process and corresponding color change. Hg^2+^ and Ag^+^ could simultaneously be detected using FRET [70]. However, this method was insufficiently sensitive for Hg^2+^ or Ag^+^ ion detection. Darbha *et al.* developed a AuNP-based sensor for the rapid, easy, and reliable detection of Hg^2+^ ions in aqueous solutions [71], which, through non-linear optical properties, had a LOD of 5 ppb (ng/mL). QDs have been utilized for FRET-based AuNPs assays for detection of environmental pollutants. An inhibition assay for identification of Pb^2+^ was developed based on the modulation in FRET efficiency between QDs and GNPs with a detection limit of 30 ppb of Pb^2+^ [72]. The positively charged QDs form FRET donor–acceptor assemblies with negatively charged GNPs by electrostatic interaction. The presence of Pb^2+^ aggregates AuNPs via an ion-templated chelation and inhibits the FRET process. A time-gated fluorescence resonance energy transfer (TGFRET) sensing strategy employing water-soluble long lifetime fluorescence quantum dots and GNPs was used to detect trace Hg^2+^ ions in aqueous solution. The sensing system exhibits the detection limits of 0.49 nM in buffer and 0.87 nM in tap water samples [73].

### 3.3. Graphene and Graphene Oxide

Fluorescent graphene-based materials have received increasing attention in recent years [74]. Their excellent biocompatibility, chemical inertness and low cytoxicity suggest them natural candidates for the detection of special targets. Through integrating the functional biomolecules, the field of graphene-based FRET biosensor targets extends from DNA to ions, small molecules, and proteins [74–79]. Chemically derived graphene oxide (GO) has traditionally served as a precursor for graphene, but is increasingly attracting researchers for its own characteristics. The intrinsic and tunable fluorescence of GO could open up exciting and previously unforeseen optical applications [75]. A fluorescence sensor was reported for the detection of Ag(I) ions based on the target-induced conformational change of a silver-specific cytosine-rich oligonucleotide (SSO) and the interactions between the fluorogenic SSO probe and graphene oxide [76]. Lee *et al.* used a platform based on chemiluminescence resonance energy transfer (CRET) between graphene nanosheets and chemiluminescent donors for homogeneous immunoassay of C-reactive protein (CRP) [77]. This graphene-based CRET platform has a LOD of 1.6 ng/mL.

Liu *et al.* developed a homogeneous competitive fluorescence-based immunoassay for rapid and sensitive detection of microcystin-LR (MC-LR) based on the assembly of colloidal grapheme and MC-LR-DNA conjugates [78]. The MC-LR-DNA fluorescence probe was quickly adsorbed onto the graphene surface through the strong noncovalent π–π stacking interactions and can be effectively quenched through FRET. The competitive binding of anti-MC-LR antibody with MC-LR-DNA destroyed the graphene/MC-LR-DNA interaction, thus resulting in the restoration of fluorescence signal. This immunosensor can be used for quantitative detection of MC-LR in water sample, with a detection limit of 0.14 μg/L. A GO-based immuno-biosensor system has been reported for the detection of rotavirus based on FRET between GO and AuNPs [79].

## 4. Optical Biosensors

### 4.1. Evanescent Wave Fiber Optic Biosensors

When light propagates through a fiber optic on the basis of total internal reflection (TIR), a thin electromagnetic field (the “evanescent wave”) generated decays exponentially with the distance from the interface with a typical penetration depth of up to several hundred nanometers [80]:

(1) $$E\left(z\right)=E_{0}\exp\left(-\delta /d_{p}\right)$$

where *δ* is the distance from the interface, the penetration depth (*d_p_*) is given by:

(2) $$d_{p}=\frac{\lambda_{ex}}{2\pi }{\left[{\left(n_{2}\right)}^{2}\sin^{2}\alpha -{\left(n_{1}\right)}^{2}\right]}^{-1/2}$$

where *λ_ex_* is the wavelength of the light, n_1_ the refractive index of the cladding region and n_2_ the refractive index of the core and *α* is the angle of incidence measured from the normal at the interface of the core and cladding. This evanescent wave can excite fluorescence in the proximity of the sensing surface, e.g., in fluorescently labeled biomolecules bound to the optical sensor surface through affinity recognition interactions. The short range of the evanescent wave enables it to discriminate between unbound and bound fluorescent complexes, hence eliminating the normally required washing procedures. Moreover, evanescent field-based waveguides are well suited for study and detection of biomolecular interaction [81].

**Figure 2.**
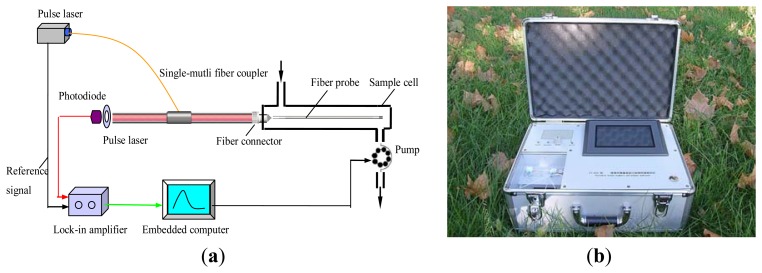
Schematic set-up of the portable evanescent wave optical fiber biosensor (EWAB): (**a**) principle scheme of the portable optical fiber biosensor and (**b**) the portable platform. Reprinted with permission from [82].

Evanescent wave fiber-optic immunosensors (EWFI) are rapid, specific, sensitive, cost effective and suitable for real-time on-site detection and have been applied to detect a wide variety of pollutants, such as TNT, 2,4-D, atrazine, *E. coli* O157:H7, and *Staphylococcal* enterotoxin B [47]. Conventional EWFI have the large size with numerous optic components (e.g., chopper, off-axis parabolic reflector, and biconvex silica lens) which makes it costly and requires crucial optical alignment restricting its use as portable device. We developed a simple, compact and portable evanescent wave all-fiber biosensor (EWAB) based on a single-multi-fiber optic coupler for simultaneous detection of 2,4,-D and MC-LR (Figure 2) [82]. With a single-multi-fiber optic coupler, both the transmission of the excitation light and the collection and transmission of fluorescence was achieved, which reduced the required optical components and alignment and resulted into a significant signal enhancement. Combination tapered fiber probes were produced by the tube-etching method and modified by covalent attachment of the MC-LR-OVA (recognition element) to a self-assembled monolayer formed onto the probe. This probe is highly resistive to non-specific binding of proteins and can be reused more than 150 times with a LOD of 0.03 μg/L and a LOD of 0.07 μg/L for MC-LR and 2,4-D, respectively [83].

Ultrasensitive DNA detection was achieved by the EWAB based on QDs and TIRF with an exceptional detection limit of 3.2 amol DNA [84]. The ssDNA coated probe was covalently immobilized onto a self-assembled alkanethiol monolayer of fiber optic probe through a streptavidin-biotinylated ssDNA strategy. A 30-mer ssDNA, the segments of the *uidA* gene of *E. coli*., was detected. The probe can be reused for more than 30 assay cycles. Based on our proposed theory, a quantitative measurement of DNA binding kinetics was achieved with high accuracy, indicating an association rate of 1.38 × 10^6^ M^−1^s^−1^ and a dissociation rate of 4.67 × 10^−3^ s^−1^. The optical biosensing platform provides a simple, cheap, fast, and robust solution for clinical diagnosis, pathology and genetics.

Mercury ions (Hg^2+^) are highly toxic and ubiquitous pollutants requiring rapid and sensitive on-site detection methods in the environment and foods. A portable, low-cost, and fast heavy metal analysis system for initial on-site/*in situ* screening of heavy metal-contaminated sites has remained a high priority to protect the environment and health. We reported an evanescent wave all-fiber optical biosensor based on structure-switching DNA for rapid on-site/*in situ* detection of heavy metal ions [85]. A DNA probe that can hybidize with a fluorescently labeled complementary DNA containing a T-T mismatch structure was covalently immobilized onto a fiber optic sensor. When the sample contains mercury ions, part of the fluorescence-labeled DNAs bind with Hg^2+^ to form T-Hg^2+^-T complexes through the folding of the DNA probe segments into a hairpin structure and dehybridization from a fiber optic sensor, resulting in a decrease in fluorescence signal. The total analysis time for a single sample, including measurement and surface regeneration, was less than 10 min with a detection limit of 1.2 nM. This sensing strategy may be an alternative method for the analysis and assessment of the transport and fate of environmental pollutants. In our previous paper [86], based on a direct structure-competitive detection mode, an evanescent wave DNA-based biosensor was also used for rapid and sensitive detection of Hg^2+^ with a detection limit of 2.1 nM. The sensor surface can be regenerated over 100 times with no significant deterioration of performance.

A proof-of-concept development of optic fiber-based immunoarray biosensor was shown for the detection of multiple small analytes [87]. Through the immobilization of two kinds of hapten conjugates (MC-LR-OVA and NB-OVA) onto the same fiber optic probe, MC-LR and TNT could be detected simultaneously and specifically within an analysis time of approximately 10 min. The LODs for MC-LR and TNT were 0.04 and 0.09 mg/L, respectively. Good regeneration performance, binding properties, and robustness of the sensor surface of the proposed immunoarray biosensor ensure the cost-effective and accurate measurement of small analytes.

### 4.2. SPR Biosensors

Surface Plasmon Resonance (SPR) is a surface-sensitive optical technique that is associated with the evanescent electromagnetic field generated on the surface of a thin metal film when excited by an incident light under total internal reflection conditions [88]. Due to the fact the evanscent field diminishes exponentially with increasing distance of penetration from the interface, SPR promotes monitoring of only surface-confined molecular interactions occurring on the transducer surface. Most of the SPR instruments use a Kretchmann configuration working at attenuated total reflectance (ATR) for excitation of surface plasmons, which can detect a small refractive index change at the metal/analyte interface, and the information of the molecular interactions can be obtained by measuring the optical intensity (or phase/polarization) of light reflected from the optical instrument. SPR biosensors allow real-time detection of minute changes in the refractive index when biorecognition molecules (e.g., antibodies) immobilized on a transducer surface bind with their biospecific targets (e.g., analytes) in solution. Since their introduction in the early 1990s, SPR biosensors have seen wide applications including clinical diagnosis, drug discovery, food analysis, environmental monitoring [10]. In general, a SPR biosensor is comprised of several important components: a light source, a detector, a transduction surface (e.g., gold-film), a prism, biorecognition molecule (e.g., antibody/antigen, DNA and aptamer) and a flow system.

The use of SPR to detect environmental contaminants, including atrazine, Dichloro-Diphenyl- Trichloroethane (DDT), 2,3,7,8-tetrachlorodibenzo-*p*-dioxin, carbaryl, 2,4-D, benzo[a]pyrene (BaP), biphenyl derivatives, and trinitrotoluene (TNT), has recently gained considerable interest [10,86–89]. An SPR immunosensor for BaP, a carcinogenic endocrine disrupting chemical, was reported to have a LOD of 10 ppt [89]. A portable SPR-based immunosensor was developed for the analysis of carbaryl in natural water samples [90]. Based on a binding inhibition immunoassay format, this immunosensor has a LOD of 1.38 μg/L. The sensor surface covalently modified by the analyte derivative allows the reuse for more than 220 regeneration cycles. The immunoassay performance of the biosensor was validated with respect to conventional high-performance liquid chromatography-mass spectrometry, and the correlation between methods was in good agreement (r^2^ > 0.998) for real water samples. Kim *et al.* [91] fabricated the sensing surface of the SPR immunosensor simply by covalent amide binding of 2,4-D-BSA conjugate on the Au-thiolate self-assembly. A LOD of 0.1 ppb 2,4-D is established with a response time of only 4 min. One of the advantages is that the immunoaffinity interactions of anti-2,4-D antibody with the 2,4-D-BSA sensor surface and 2,4-D in solution could be significantly modulated by the control immobilization of 2,4-D-BSA on the SAM surface. As a result, the sensitivity of the SPR immunosensor is enhanced by about 10-fold to 10 ppt without using any high-molecular-weight labels. Localized surface plasmon resonance (LSPR) effect using AuNP for signal amplification was also investigated [92]. The amplification method of indirect competitive inhibition and LSPR were integrated for the fabrication of an immunosurface using AuNP. The detection range of TNT using this immunosurface was from 10 ppt to 100 ppb.

### 4.3. Nano-Structured Optical Biosensors

Progress in nanotechnology, microelectronics and microfluidics could facilitate development of miniaturized, rapid, ultrasensitive and inexpensive nano-structured optical biosensing platforms for rapid toxicity screening and multianalyte testing. These devices are likely to become more compact, robust, smaller and adaptable for in-field and continuous field-based environmental monitoring monitoring. A fiber-optic nanosensor was designed with taper optical fibers, onto which biorecognition molecules (e.g., antibody, peptides, and nucleic acids) was immobilized. This sensor can probe individual chemical species in a living cell [93]. *In situ* measurements of the carcinogen BaP in a single cell could be achieved by a fiber-optic immuno-nanosensor [94], and the quantitative detection ranges from 1.56 × 10^−1^ M to 1.56 × 10^−8^ M.

A mesoporous silica nanosensor, which responds selectively to Fe^2+^ (pH = 8) and Cu^2+^ (pH = 12) with a distinguishable colour change perceivable by the naked eye with a detection limit of approximately 50 ppb was synthesized by the co-condensation method [95]. A whispering gallery mode (WGM) nanosensor consists of an optical resonator and a circular cavity. A tapered optical fiber placed next to the cavity was used to introduce light evanescent coupling. Armani *et al.* developed a WGM nanobiosensor using a micro-toroid cavity with a Q greater than [96]. In this study, single-molecule detection sensitivity for antibody-antigen binding was demonstrated. This nanobiosensor could perform real time single-molecule detection and exhibited a dynamic range from 5 aM to 1 μM.

## 5. Optical Biosensors for Pollution Control and Early-Warning

The increasing number of pollutants and their derivatives both in surface and ground waters as well as the stricter regulations for pollutant detection set by legislative bodies prompted great interest in a cheap general network system for pollution control and early warning [97]. An early warning system (EWS) is an integrated system for monitoring, analyzing, interpreting, and communicating monitoring data, in which the continuous real-time detection is often performed using sensors/biosensors and a generic warning or trigger an alarm is provided when a contaminant is detected in the water [98]. The EWS identifies low probability/high-impact contamination events in sufficient time to safeguard public health.

The ideal integrated EWS must provide a rapid response and warning in sufficient time for action, automatic sampling and automation detection, sufficient sensitivity, and minimal false-positives/false-negatives [97]. Optical biosensors have been proven to outperform other types of sensors in multitarget sensing and continuous real-time on-site monitoring [11]. Therefore, optical biosensors have been integrated into many EWSs for the mapping of contamination from accidental spills or pollution events.

Biosensors based on luminescent bacteria are valuable tools for the online monitoring and early warning for surface and drinking water. Many bacterial strains have been described for the detection of a broad range of toxicity parameters such as organic pollutants and heavy metals. A multi-channel bioluminescent bacterial biosensor has been developed for the online detection of metals and toxicity [99]. Using a set of four bioluminescent bacteria (*E. coli* DH1 pBzntlux, pBarslux, pBcoplux, and *E. coli* XL1 pBfiluxCDABE), 0.5 μM CdCl_2_ and 5 μM As_2_O_3_ from an influent were detected online. This biosensor demonstrated the simultaneous on-line cross detection of one or several heavy metals as well as the measurement of the overall toxicity of the sample.

The bbe Algae Toximeter continuously determines toxic substances in water based on changes in the fluorescence spectrum and fluorescence kinetics of the algae [100]. During the test, the standardized algae, automatically and independently cultivated, are added to the water sample, after which the active chlorophyll concentration is analyzed. If the algae are damaged, such as through herbicides that reduce activity or indirectly through oxygen evolution, an alarm is induced. This system has high sensitivity in recognizing herbicides and their by-products to achieve a higher temporal resolution of the monitored water.

Freshly cultivated *Vibrio fischeri* bacteria were used as a biological sensor in the TOX control system [101]. Luminescence is measured before and after exposition to calculate the percent inhibition. The increasing toxicity of the sample resulted in the greater light loss from the test suspension of luminescent bacteria. This system combines the advantages of whole organism toxicity testing and instrumental precision.

An automated water analyzer computer-supported system (AWACSS) based on an optical immunoassay technology has been developed, which can rapidly measure several trace organic pollutants without any prior sample pre-treatment [102]. The AWACSS is an early-warning system utilizing a network of measurement and control stations. The system consists of four major components: the AWACSS instrument with fluidics control and optical transducer chip, the HTC PAL auto-sampler for sample preparation, the personal computer at the sampling site, and the server with database and Web site. This system had been applied for the rapid detection of multi-targets (e.g., estrone, propanil, isoproturon, atrazine, bisphenol A, sulphonamides, and progesterone). Detection limits of most targets were in a few nanogram or even sub-nanogram per litre range, while its selectivity allowed for trace analysis even in complex matrices. A Web-based AWACSS system enables internet-based networking between the measurement and control stations, global management, trend analysis, and early-warning applications.

In our recent study, an innovative automated online optical biosensing system (AOBS) was developed for the rapid detection and early warning of microcystin-LR (MC-LR) [103]. With an indirect competitive detection mode, samples containing different concentrations of MC-LR were premixed with a certain concentration of fluorescence-labeled anti-MC-LR-MAb, which binds to MC-LR with high specificity. Then, the sample mixture is pumped onto the biochip surface modified by MC-LR-ovalbumin, and a higher concentration of MC-LR led to less fluorescence-labeled antibody bound onto the biochip surface and thus to lower fluorescence signal. The quantification of MC-LR ranges from 0.2 μg/L to 4 μg/L and the LOD is 0.09 μg/L. This system has successfully been applied to long-term, continuous determination and early-warning for MC-LR in Lake Tai (China) about one year. As the biochip contains six sensing points, the AOBS allows the simultaneous determination of six different pollutants in environmental matrices when each point is modified by other analyte conjugates using their fluorescence-labeled antibodies. The AOBS paves the way for a vital routine online analysis that satisfies the high demand for ensuring the safety of drinking water sources. The AOBS can also serve as early warning system for accidental or intentional water pollution.

## 6. Key Trends and Perspectives

The recent progress in optical biosensor technology has revolutionized our ability to characterize and quantify environmental pollutants, and undoubtedly offers benefits for environmental pollution control and early warning [2–6,104]. Optical biosensors have several significant advantages for such applications [4–12,104]: (1) the optical biosensor provides a rapid, simple, and sensitive, and selective assay method; (2) long-period, automated high-frequency measurements of environmental pollutants will become possible; (3) novel nano-materials and functional biomaterials may offer unique properties for real-time *in-situ* assays of binding kinetics between environmental pollutants and functional biomolecules; (4) biosensing arrays enable the development of more compact, robust, smaller, and adaptable optical biosensors for rapid toxicity screening and multi-analyte testing of the environmental pollutants; (5) integration of microelectronics and microfluidics into optical biosensors will miniaturize optical biorecognition elements; (6) wireless-communication technology facilitates the emergence of environmental sensor networks; (7) the long-term, high-frequency online detection ability of biosensors can provide new insight into the production and migration mechanism and fate of environmental pollutants in combination with physicochemical parameters (such as temperature, pH). Although various challenges still remains in creating improved, cost-effective, and more reliable biosensors [11], optical biosensors will provide the most productive paths for environmental pollution control and early warning.

## Figures and Tables

**Table 1.** A listing of DNA/RNA aptamers recently reported in the open literature that have been confirmed to bind to environmental pollutants. The dissociation constant (K_d_), a measurement of binding affinity, is included, as well as the year of aptamer development.

**No**	**Target**	**Aptamer Type**	**Binding Affinity(K_d_)**	**Year**	**Ref.**
1	Polychlorinated biphenyls (PCB77)	DNA	4.02, 8.32 μM	2012	[27]
2	Polychlorinated biphenyls (PCB72 and PCB106)	DNA	60–100 nM	2012	[28]
3	Organophosphorus compounds (pesticides:phorate,profenofos, isocarbophos, omethoateas)	DNA	0.8–2.5 μM	2012	[29]
4	Bisphenol A	DNA	8.3 nM	2011	[30]
5	17*β*-Estradiol	DNA	0.13 μM	2007	[31]
6	Chloramphenicol	DNA	0.8 and 1 μM	2011	[32]
7	Oxytetracycline	DNA	10 nM	2008	[33]
8	Tetracycline	DNA	64 nM	2008	[33]
9	Kanamycin	DNA	78.8 nM	2011	[35]
10	Ampicillin	DNA	9.4–13.4 nM	2012	[36]
11	Ochratoxin A	DNA	96–293 nM	2011	[37]
12	*E. coli*	DNA	No shown	2010	[38]
13	*Staphylococcus aureus* Enterotoxin B	DNA	No shown	2012	[39]
14	Phenylphosphonic dichloride	DNA	>50 μM	2011	[40]
15	Arsenic	DNA	4.95–7.05 nM	2009	[41]
16	Microcystins	DNA	50 nM	2012	[42]
17	Atrazine	RNA	2 μM	2010	[43]
18	Tobramycin	RNA	16 μM	2007	[44]
